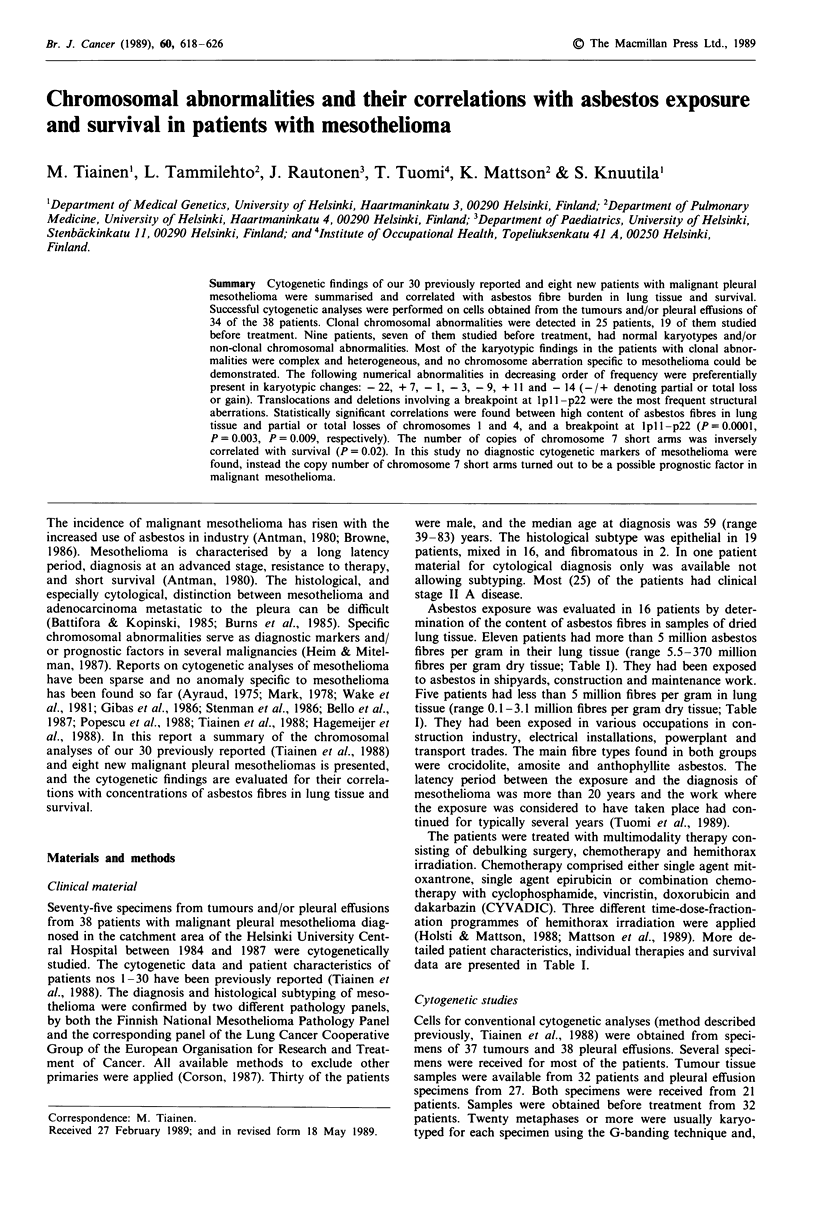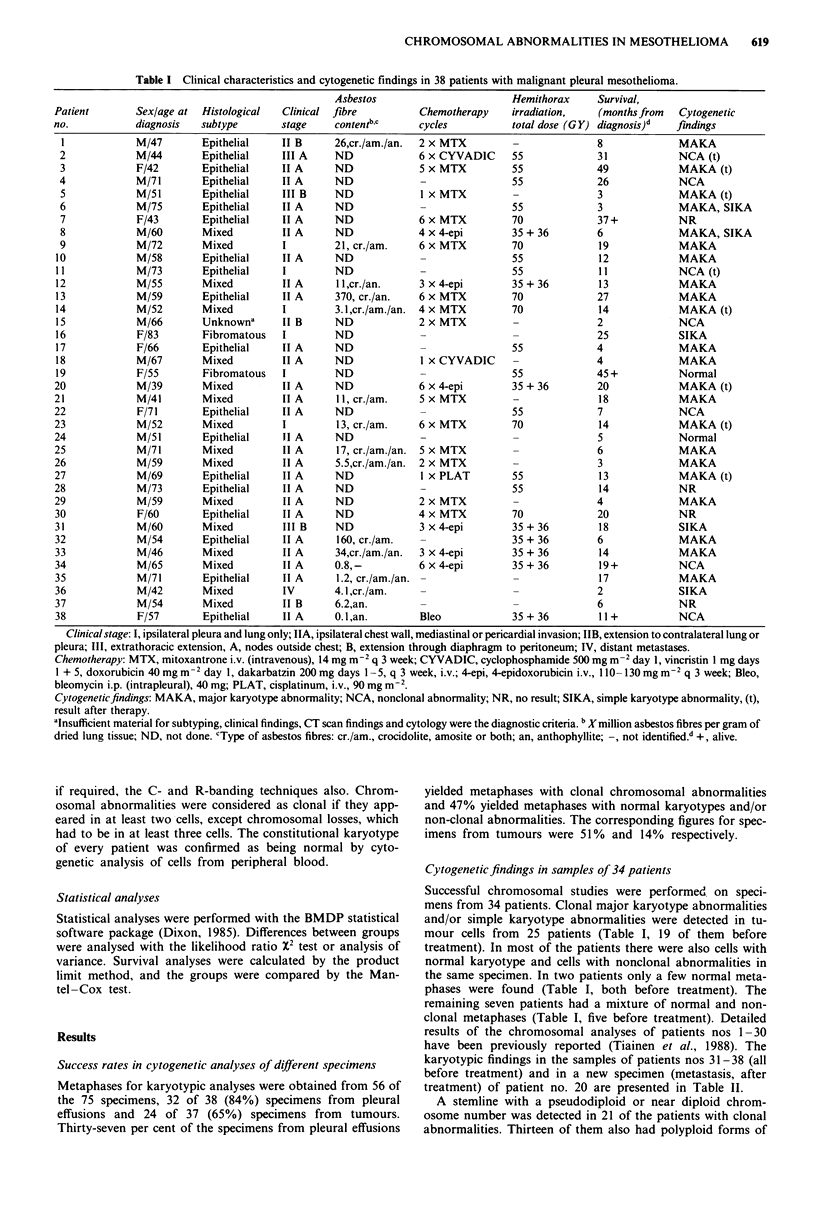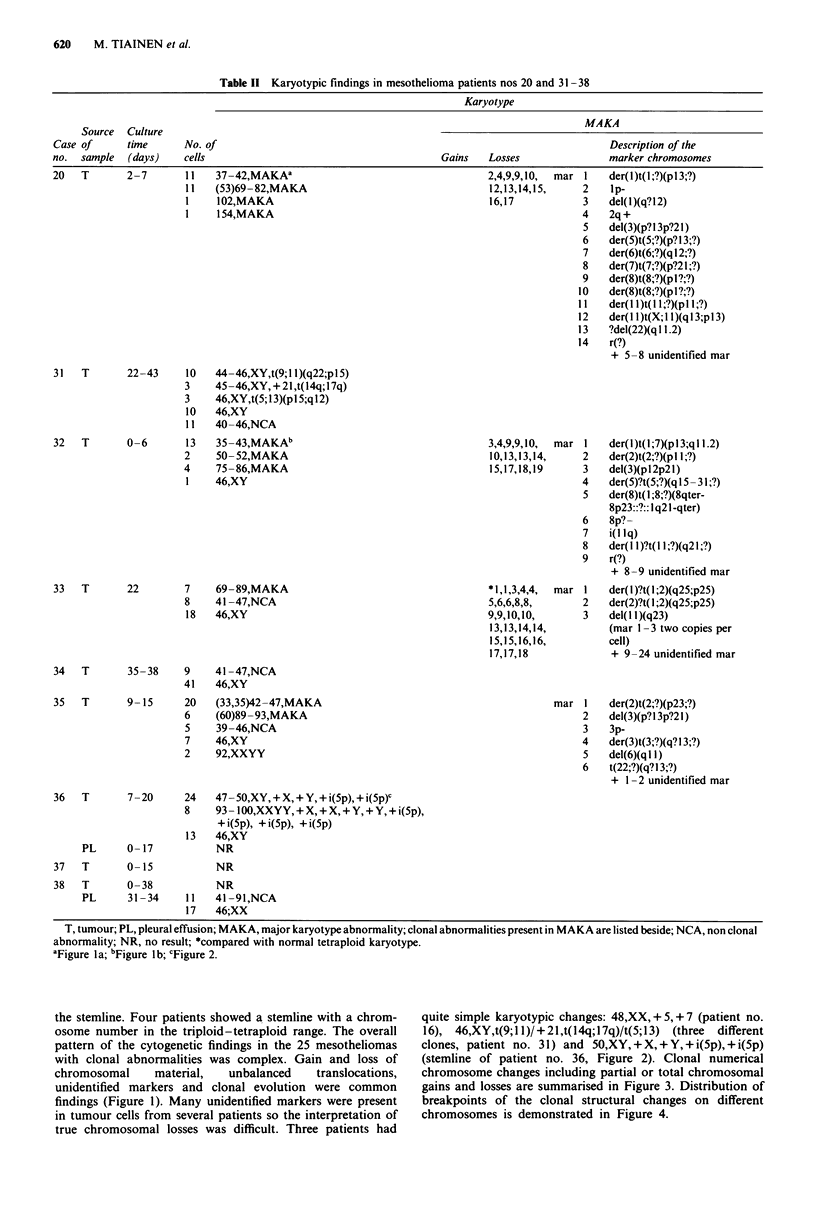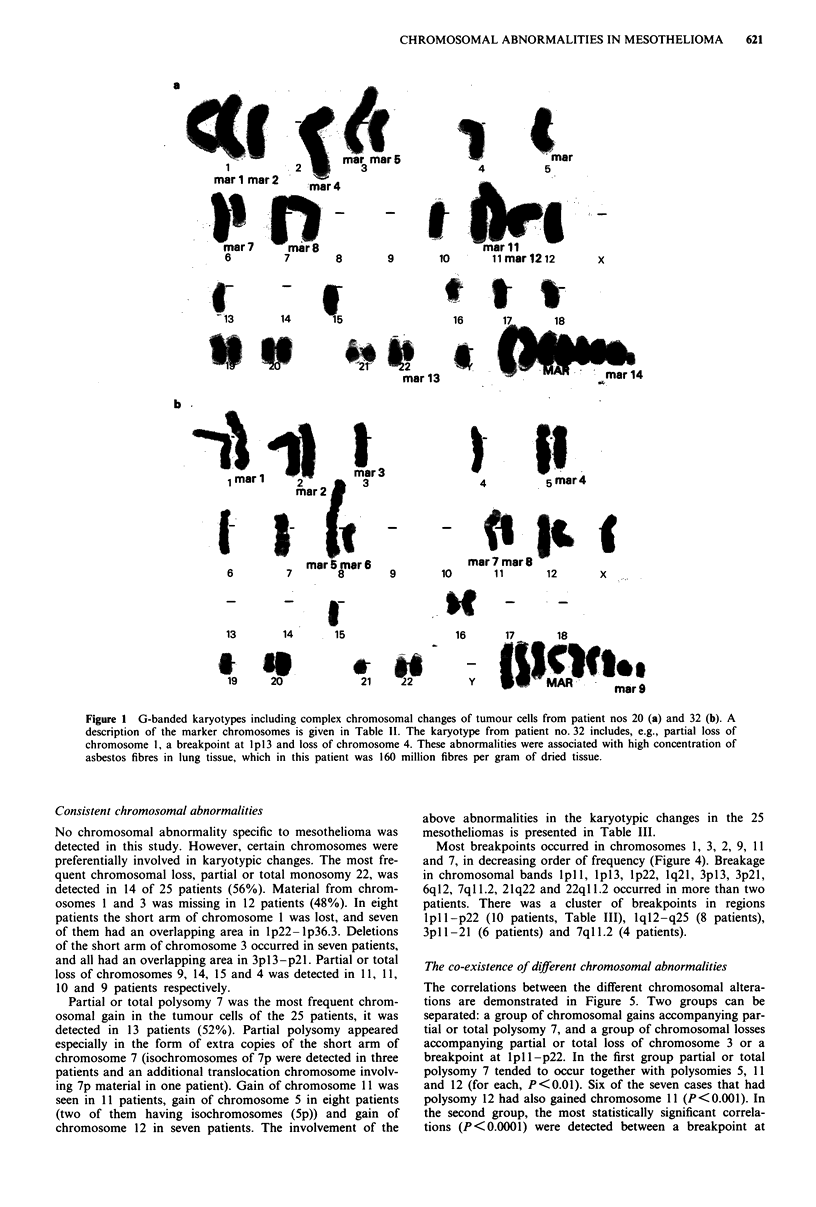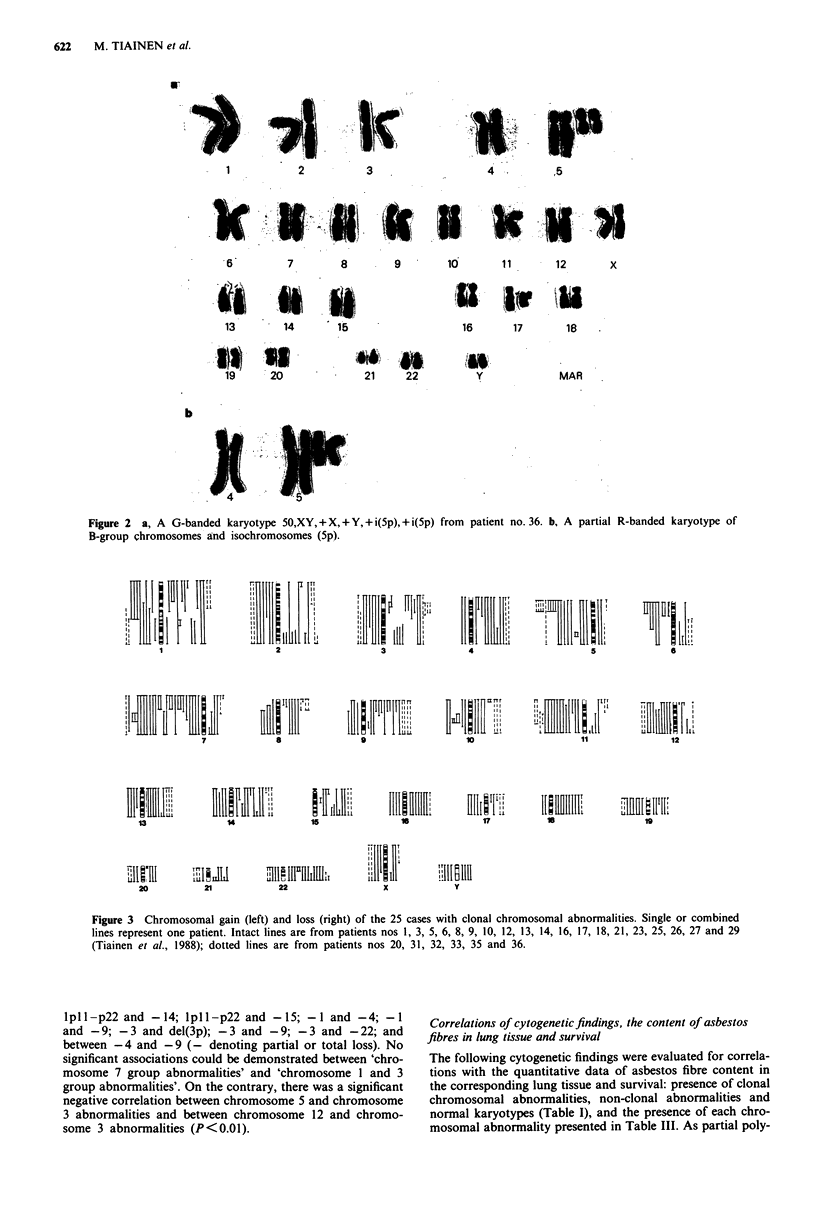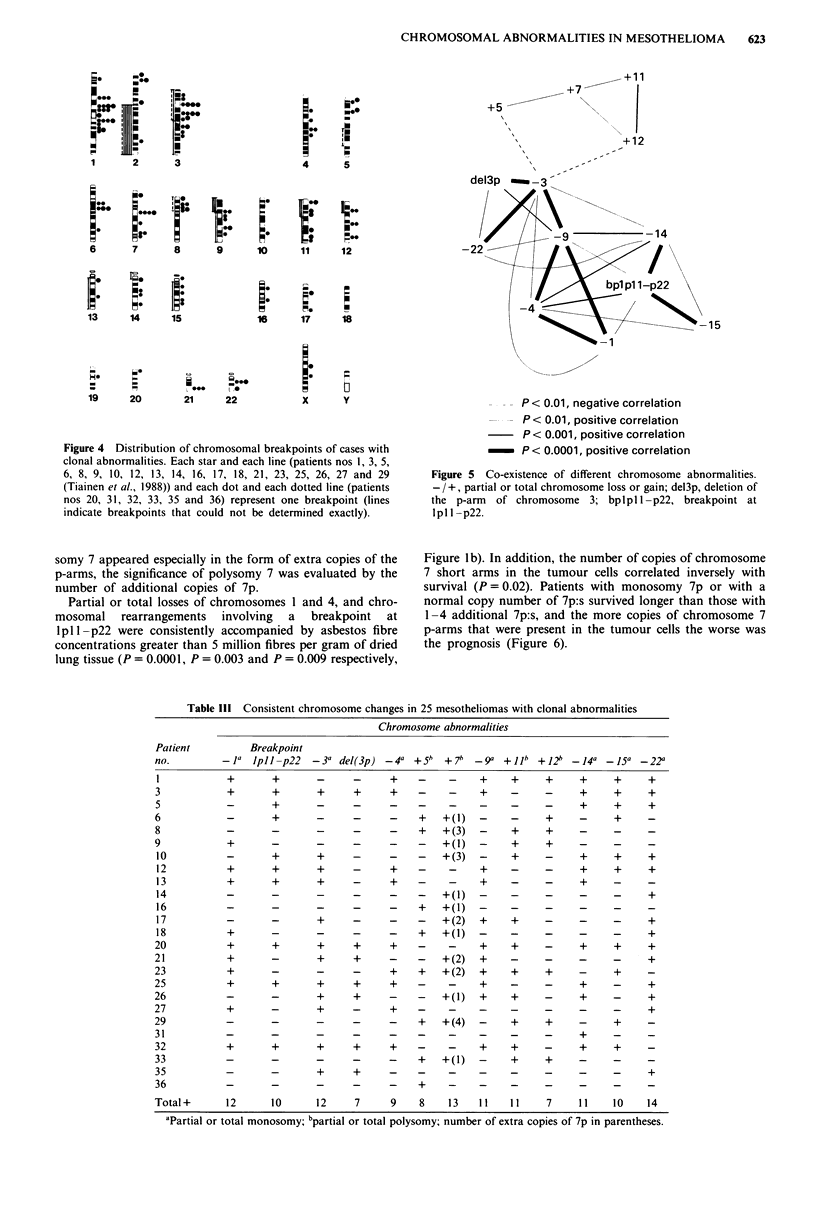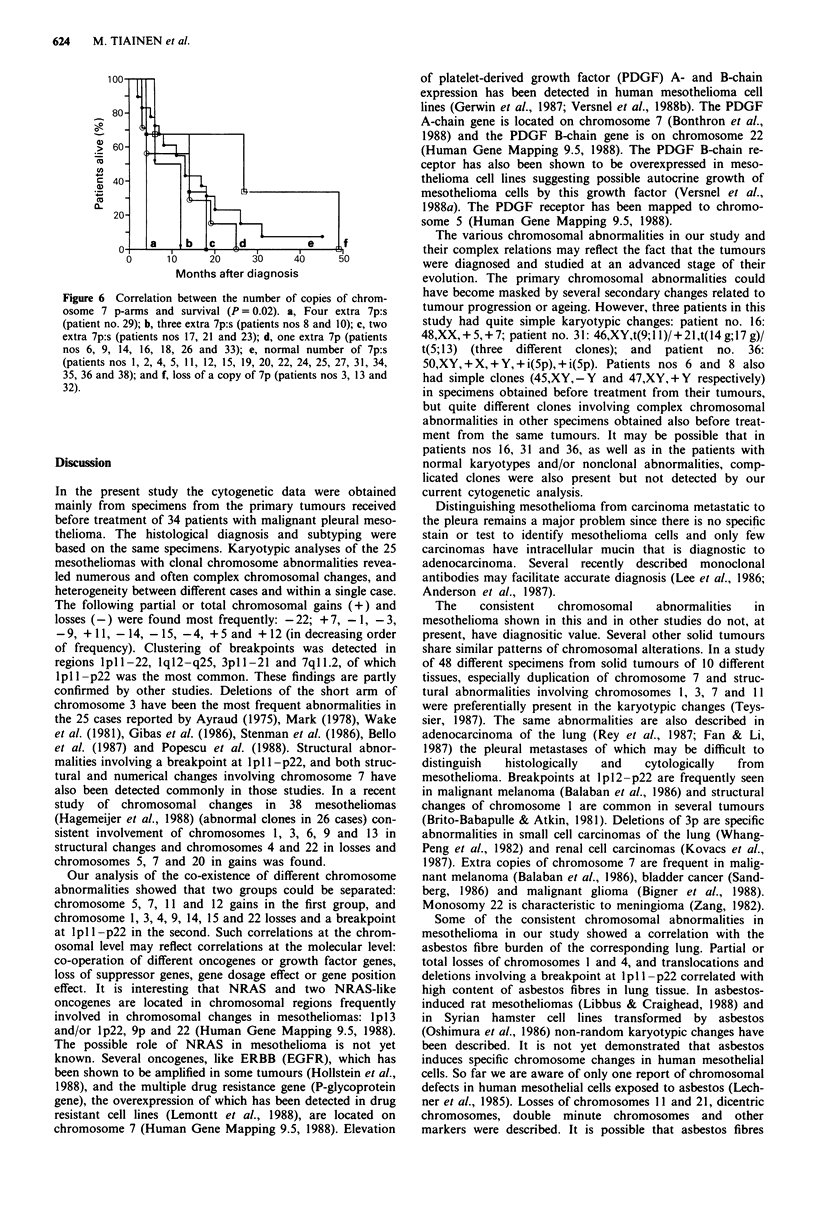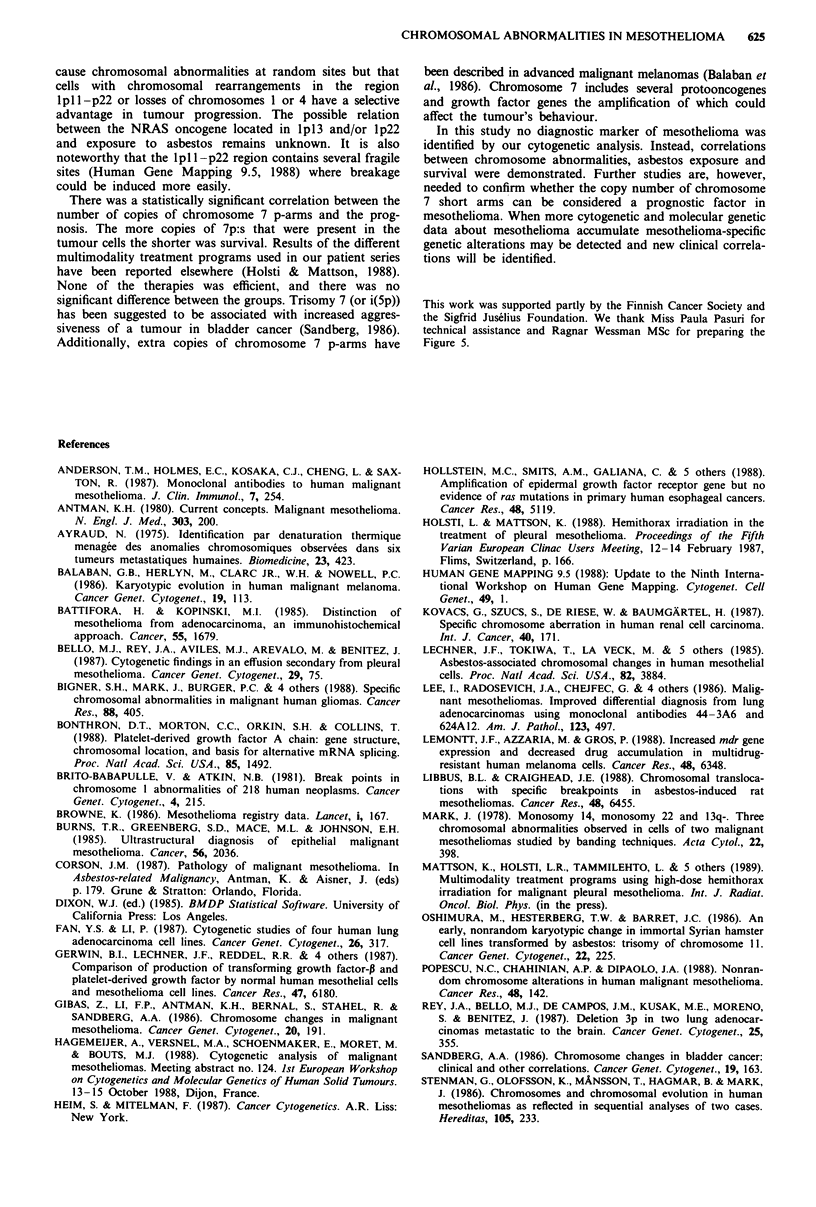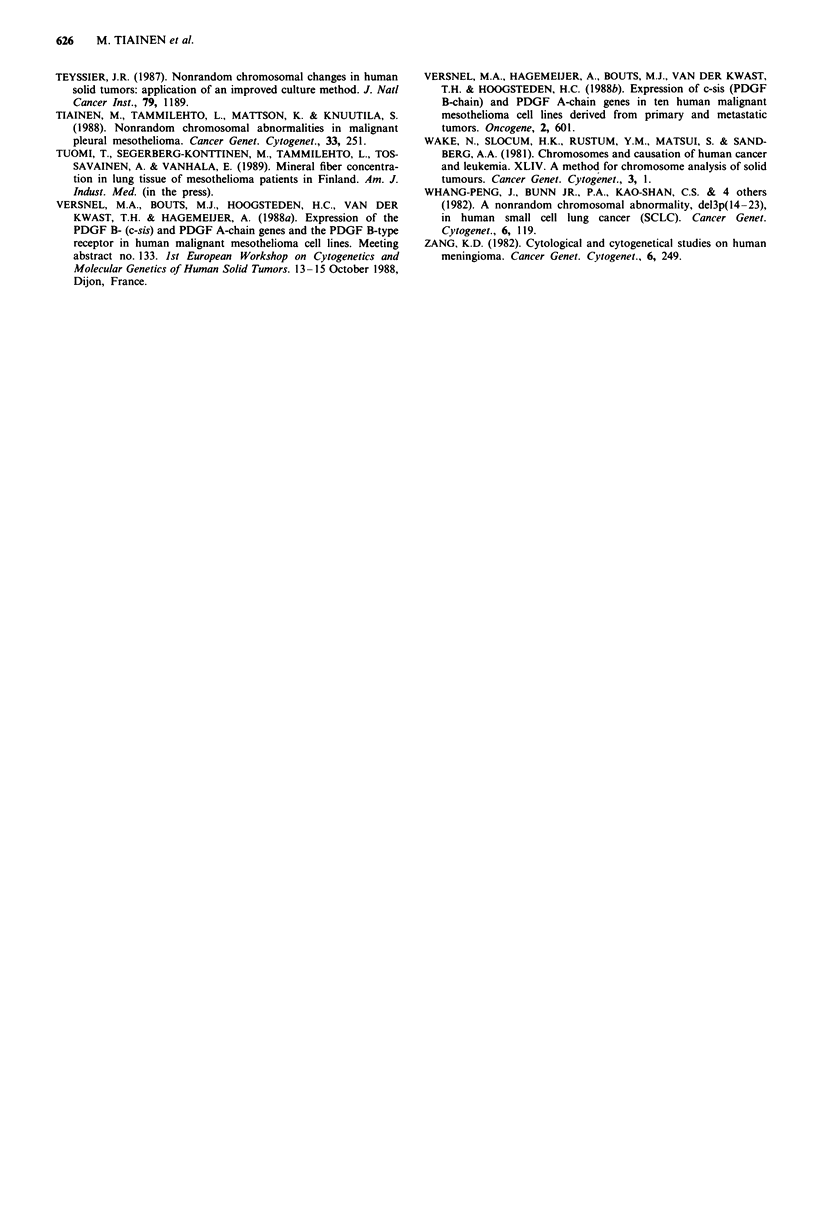# Chromosomal abnormalities and their correlations with asbestos exposure and survival in patients with mesothelioma.

**DOI:** 10.1038/bjc.1989.326

**Published:** 1989-10

**Authors:** M. Tiainen, L. Tammilehto, J. Rautonen, T. Tuomi, K. Mattson, S. Knuutila

**Affiliations:** Department of Medical Genetics, University of Helsinki, Finland.

## Abstract

**Images:**


Br. J. Cancer (1989), 60, 618-626 © The Macmillan Press Ltd., 1989

### Chromosomal abnormalities and their correlations with asbestos exposure and survival in patients with mesothelioma

M. Tiainen[1], L. Tammilehto[2], J. Rautonen[3], T. Tuomi[4], K. Mattson[2] & S. Knuutila[1]

[1]*Department of Medical Genetics, University of Helsinki, Haartmaninkatu 3, 00290 Helsinki, Finland;* [2]*Department of Pulmonary Medicine, University of Helsinki, Haartmaninkatu 4, 00290 Helsinki, Finland;* [3]*Department of Paediatrics, University of Helsinki, Stenbäckinkatu 11, 00290 Helsinki, Finland; and* [4]*Institute of Occupational Health, Topeliuksenkatu 41 A, 00250 Helsinki, Finland.*

**Summary** Cytogenetic findings of our 30 previously reported and eight new patients with malignant pleural mesothelioma were summarised and correlated with asbestos fibre burden in lung tissue and survival. Successful cytogenetic analyses were performed on cells obtained from the tumours and/or pleural effusions of 34 of the 38 patients. Clonal chromosomal abnormalities were detected in 25 patients, 19 of them studied before treatment. Nine patients, seven of them studied before treatment, had normal karyotypes and/or non-clonal chromosomal abnormalities. Most of the karyotypic findings in the patients with clonal abnormalities were complex and heterogeneous, and no chromosome aberration specific to mesothelioma could be demonstrated. The following numerical abnormalities in decreasing order of frequency were preferentially present in karyotypic changes: − 22, + 7, − 1, − 3, − 9, + 11 and − 14 (−/+ denoting partial or total loss or gain). Translocations and deletions involving a breakpoint at 1p11−p22 were the most frequent structural aberrations. Statistically significant correlations were found between high content of asbestos fibres in lung tissue and partial or total losses of chromosomes 1 and 4, and a breakpoint at 1p11−p22 ($P = 0.0001$, $P = 0.003$, $P = 0.009$, respectively). The number of copies of chromosome 7 short arms was inversely correlated with survival ($P = 0.02$). In this study no diagnostic cytogenetic markers of mesothelioma were found, instead the copy number of chromosome 7 short arms turned out to be a possible prognostic factor in malignant mesothelioma.

The incidence of malignant mesothelioma has risen with the increased use of asbestos in industry (Antman, 1980; Browne, 1986). Mesothelioma is characterised by a long latency period, diagnosis at an advanced stage, resistance to therapy, and short survival (Antman, 1980). The histological, and especially cytological, distinction between mesothelioma and adenocarcinoma metastatic to the pleura can be difficult (Battifora & Kopinski, 1985; Burns *et al.*, 1985). Specific chromosomal abnormalities serve as diagnostic markers and/or prognostic factors in several malignancies (Heim & Mitelman, 1987). Reports on cytogenetic analyses of mesothelioma have been sparse and no anomaly specific to mesothelioma has been found so far (Ayraud, 1975; Mark, 1978; Wake *et al.*, 1981; Gibas *et al.*, 1986; Stenman *et al.*, 1986; Bello *et al.*, 1987; Popescu *et al.*, 1988; Tiainen *et al.*, 1988; Hagemeijer *et al.*, 1988). In this report a summary of the chromosomal analyses of our 30 previously reported (Tiainen *et al.*, 1988) and eight new malignant pleural mesotheliomas is presented, and the cytogenetic findings are evaluated for their correlations with concentrations of asbestos fibres in lung tissue and survival.

### Materials and methods

### *Clinical material*

Seventy-five specimens from tumours and/or pleural effusions from 38 patients with malignant pleural mesothelioma diagnosed in the catchment area of the Helsinki University Central Hospital between 1984 and 1987 were cytogenetically studied. The cytogenetic data and patient characteristics of patients nos 1−30 have been previously reported (Tiainen *et al.*, 1988). The diagnosis and histological subtyping of mesothelioma were confirmed by two different pathology panels, by both the Finnish National Mesothelioma Pathology Panel and the corresponding panel of the Lung Cancer Cooperative Group of the European Organisation for Research and Treatment of Cancer. All available methods to exclude other primaries were applied (Corson, 1987). Thirty of the patients were male, and the median age at diagnosis was 59 (range 39−83) years. The histological subtype was epithelial in 19 patients, mixed in 16, and fibromatous in 2. In one patient material for cytological diagnosis only was available not allowing subtyping. Most (25) of the patients had clinical stage II A disease.

Asbestos exposure was evaluated in 16 patients by determination of the content of asbestos fibres in samples of dried lung tissue. Eleven patients had more than 5 million asbestos fibres per gram in their lung tissue (range 5.5−370 million fibres per gram dry tissue; Table I). They had been exposed to asbestos in shipyards, construction and maintenance work. Five patients had less than 5 million fibres per gram in lung tissue (range 0.1−3.1 million fibres per gram dry tissue; Table I). They had been exposed in various occupations in construction industry, electrical installations, powerplant and transport trades. The main fibre types found in both groups were crocidolite, amosite and anthophyllite asbestos. The latency period between the exposure and the diagnosis of mesothelioma was more than 20 years and the work where the exposure was considered to have taken place had continued for typically several years (Tuomi *et al.*, 1989).

The patients were treated with multimodality therapy consisting of debulking surgery, chemotherapy and hemithorax irradiation. Chemotherapy comprised either single agent mitoxantrone, single agent epirubicin or combination chemotherapy with cyclophosphamide, vincristin, doxorubicin and dakarbazin (CYVADIC). Three different time-dose-fractionation programmes of hemithorax irradiation were applied (Holsti & Mattson, 1988; Mattson *et al.*, 1989). More detailed patient characteristics, individual therapies and survival data are presented in Table I.

### *Cytogenetic studies*

Cells for conventional cytogenetic analyses (method described previously, Tiainen *et al.*, 1988) were obtained from specimens of 37 tumours and 38 pleural effusions. Several specimens were received for most of the patients. Tumour tissue samples were available from 32 patients and pleural effusion specimens from 27. Both specimens were received from 21 patients. Samples were obtained before treatment from 32 patients. Twenty metaphases or more were usually karyotyped for each specimen using the G-banding technique and,

Correspondence: M. Tiainen.
Received 27 February 1989; and in revised form 18 May 1989.

**Table I** Clinical characteristics and cytogenetic findings in 38 patients with malignant pleural mesothelioma.

| *Patient no.* | *Sex/age at diagnosis* | *Histological subtype* | *Clinical stage* | *Asbestos fibre content*[b,c] | *Chemotherapy cycles* | *Hemithorax irradiation, total dose (GY)* | *Survival, (months from diagnosis)*[d] | *Cytogenetic findings* |
|---|---|---|---|---|---|---|---|---|
| 1 | M/47 | Epithelial | II B | 26,cr./am./an. | 2 × MTX | – | 8 | MAKA |
| 2 | M/44 | Epithelial | III A | ND | 6 × CYVADIC | 55 | 31 | NCA (t) |
| 3 | F/42 | Epithelial | II A | ND | 5 × MTX | 55 | 49 | MAKA (t) |
| 4 | M/71 | Epithelial | II A | ND | – | 55 | 26 | NCA |
| 5 | M/51 | Epithelial | III B | ND | 1 × MTX | – | 3 | MAKA (t) |
| 6 | M/75 | Epithelial | II A | ND | – | 55 | 3 | MAKA, SIKA |
| 7 | F/43 | Epithelial | II A | ND | 6 × MTX | 70 | 37+ | NR |
| 8 | M/60 | Mixed | II A | ND | 4 × 4-epi | 35 + 36 | 6 | MAKA, SIKA |
| 9 | M/72 | Mixed | I | 21, cr./am. | 6 × MTX | 70 | 19 | MAKA |
| 10 | M/58 | Epithelial | II A | ND | – | 55 | 12 | MAKA |
| 11 | M/73 | Epithelial | I | ND | – | 55 | 11 | NCA (t) |
| 12 | M/55 | Mixed | II A | 11,cr./an. | 3 × 4-epi | 35 + 36 | 13 | MAKA |
| 13 | M/59 | Epithelial | II A | 370, cr./an. | 6 × MTX | 70 | 27 | MAKA |
| 14 | M/52 | Mixed | I | 3.1,cr./am./an. | 4 × MTX | 70 | 14 | MAKA (t) |
| 15 | M/66 | Unknown[a] | II B | ND | 2 × MTX | – | 2 | NCA |
| 16 | F/83 | Fibromatous | I | ND | – | – | 25 | SIKA |
| 17 | F/66 | Epithelial | II A | ND | – | 55 | 4 | MAKA |
| 18 | M/67 | Mixed | II A | ND | 1 × CYVADIC | – | 4 | MAKA |
| 19 | F/55 | Fibromatous | I | ND | – | 55 | 45+ | Normal |
| 20 | M/39 | Mixed | II A | ND | 6 × 4-epi | 35 + 36 | 20 | MAKA (t) |
| 21 | M/41 | Mixed | II A | 11, cr./am. | 5 × MTX | – | 18 | MAKA |
| 22 | F/71 | Epithelial | II A | ND | – | 55 | 7 | NCA |
| 23 | M/52 | Mixed | I | 13, cr./am. | 6 × MTX | 70 | 14 | MAKA (t) |
| 24 | M/51 | Epithelial | II A | ND | – | – | 5 | Normal |
| 25 | M/71 | Mixed | II A | 17, cr./am./an. | 5 × MTX | – | 6 | MAKA |
| 26 | M/59 | Mixed | II A | 5.5,cr./am./an. | 2 × MTX | – | 3 | MAKA |
| 27 | M/69 | Epithelial | II A | ND | 1 × PLAT | 55 | 13 | MAKA (t) |
| 28 | M/73 | Epithelial | II A | ND | – | 55 | 14 | NR |
| 29 | M/59 | Mixed | II A | ND | 2 × MTX | – | 4 | MAKA |
| 30 | F/60 | Epithelial | II A | ND | 4 × MTX | 70 | 20 | NR |
| 31 | M/60 | Mixed | III B | ND | 3 × 4-epi | 35 + 36 | 18 | SIKA |
| 32 | M/54 | Epithelial | II A | 160, cr./am. | – | 35 + 36 | 6 | MAKA |
| 33 | M/46 | Mixed | II A | 34,cr./am./an. | 3 × 4-epi | 35 + 36 | 14 | MAKA |
| 34 | M/65 | Mixed | II A | 0.8,– | 6 × 4-epi | 35 + 36 | 19+ | NCA |
| 35 | M/71 | Epithelial | II A | 1.2, cr./am./an. | – | – | 17 | MAKA |
| 36 | M/42 | Mixed | IV | 4.1,cr./am. | – | – | 2 | SIKA |
| 37 | M/54 | Mixed | II B | 6.2,an. | – | – | 6 | NR |
| 38 | F/57 | Epithelial | II A | 0.1,an. | Bleo | 35 + 36 | 11+ | NCA |

*Clinical stage*: I, ipsilateral pleura and lung only; IIA, ipsilateral chest wall, mediastinal or pericardial invasion; IIB, extension to contralateral lung or pleura; III, extrathoracic extension, A, nodes outside chest; B, extension through diaphragm to peritoneum; IV, distant metastases.
*Chemotherapy*: MTX, mitoxantrone i.v. (intravenous), 14 mg $m^{-2}$ q 3 week; CYVADIC, cyclophosphamide 500 mg $m^{-2}$ day 1, vincristin 1 mg days 1 + 5, doxorubicin 40 mg $m^{-2}$ day 1, dakarbatzin 200 mg days 1–5, q 3 week, i.v.; 4-epi, 4-epidoxorubicin i.v., 110–130 mg $m^{-2}$ q 3 week; Bleo, bleomycin i.p. (intrapleural), 40 mg; PLAT, cisplatinum, i.v., 90 mg $m^{-2}$.
*Cytogenetic findings*: MAKA, major karyotype abnormality; NCA, nonclonal abnormality; NR, no result; SIKA, simple karyotype abnormality, (t), result after therapy.
[a]Insufficient material for subtyping, clinical findings, CT scan findings and cytology were the diagnostic criteria. [b] *X* million asbestos fibres per gram of dried lung tissue; ND, not done. [c]Type of asbestos fibres: cr./am., crocidolite, amosite or both; an, anthophyllite; –, not identified.[d] +, alive.

if required, the C- and R-banding techniques also. Chromosomal abnormalities were considered as clonal if they appeared in at least two cells, except chromosomal losses, which had to be in at least three cells. The constitutional karyotype of every patient was confirmed as being normal by cytogenetic analysis of cells from peripheral blood.

### *Statistical analyses*

Statistical analyses were performed with the BMDP statistical software package (Dixon, 1985). Differences between groups were analysed with the likelihood ratio $\chi^2$ test or analysis of variance. Survival analyses were calculated by the product limit method, and the groups were compared by the Mantel–Cox test.

### Results

### *Success rates in cytogenetic analyses of different specimens*

Metaphases for karyotypic analyses were obtained from 56 of the 75 specimens, 32 of 38 (84%) specimens from pleural effusions and 24 of 37 (65%) specimens from tumours. Thirty-seven per cent of the specimens from pleural effusions yielded metaphases with clonal chromosomal abnormalities and 47% yielded metaphases with normal karyotypes and/or non-clonal abnormalities. The corresponding figures for specimens from tumours were 51% and 14% respectively.

### *Cytogenetic findings in samples of 34 patients*

Successful chromosomal studies were performed on specimens from 34 patients. Clonal major karyotype abnormalities and/or simple karyotype abnormalities were detected in tumour cells from 25 patients (Table I, 19 of them before treatment). In most of the patients there were also cells with normal karyotype and cells with nonclonal abnormalities in the same specimen. In two patients only a few normal metaphases were found (Table I, both before treatment). The remaining seven patients had a mixture of normal and nonclonal metaphases (Table I, five before treatment). Detailed results of the chromosomal analyses of patients nos 1–30 have been previously reported (Tiainen *et al.*, 1988). The karyotypic findings in the samples of patients nos 31–38 (all before treatment) and in a new specimen (metastasis, after treatment) of patient no. 20 are presented in Table II.

A stemline with a pseudodiploid or near diploid chromosome number was detected in 21 of the patients with clonal abnormalities. Thirteen of them also had polyploid forms of

**Table II** Karyotypic findings in mesothelioma patients nos 20 and 31–38

| Case no. | Source of sample | Culture time (days) | No. of cells | Karyotype | MAKA Gains | MAKA Losses | | MAKA Description of the marker chromosomes |
|---|---|---|---|---|---|---|---|---|
| 20 | T | 2–7 | 11 | 37–42,MAKA[a] | | 2,4,9,9,10, | mar 1 | der(1)t(1;?)(p13;?) |
| | | | 11 | (53)69–82,MAKA | | 12,13,14,15, | 2 | 1p- |
| | | | 1 | 102,MAKA | | 16,17 | 3 | del(1)(q?12) |
| | | | 1 | 154,MAKA | | | 4 | 2q+ |
| | | | | | | | 5 | del(3)(p?13p?21) |
| | | | | | | | 6 | der(5)t(5;?)(p?13;?) |
| | | | | | | | 7 | der(6)t(6;?)(q12;?) |
| | | | | | | | 8 | der(7)t(7;?)(p?21;?) |
| | | | | | | | 9 | der(8)t(8;?)(p1?;?) |
| | | | | | | | 10 | der(8)t(8;?)(p1?;?) |
| | | | | | | | 11 | der(11)t(11;?)(p11;?) |
| | | | | | | | 12 | der(11)t(X;11)(q13;p13) |
| | | | | | | | 13 | ?del(22)(q11.2) |
| | | | | | | | 14 | r(?) |
| | | | | | | | | + 5–8 unidentified mar |
| 31 | T | 22–43 | 10 | 44–46,XY,t(9;11)(q22;p15) | | | | |
| | | | 3 | 45–46,XY,+21,t(14q;17q) | | | | |
| | | | 3 | 46,XY,t(5;13)(p15;q12) | | | | |
| | | | 10 | 46,XY | | | | |
| | | | 11 | 40–46,NCA | | | | |
| 32 | T | 0–6 | 13 | 35–43,MAKA[b] | | 3,4,9,9,10, | mar 1 | der(1)t(1;7)(p13;q11.2) |
| | | | 2 | 50–52,MAKA | | 10,13,13,14, | 2 | der(2)t(2;?)(p11;?) |
| | | | 4 | 75–86,MAKA | | 15,17,18,19 | 3 | del(3)(p12p21) |
| | | | 1 | 46,XY | | | 4 | der(5)?t(5;?)(q15–31;?) |
| | | | | | | | 5 | der(8)t(1;8;?)(8qter-8p23::?::1q21-qter) |
| | | | | | | | 6 | 8p?- |
| | | | | | | | 7 | i(11q) |
| | | | | | | | 8 | der(11)?t(11;?)(q21;?) |
| | | | | | | | 9 | r(?) |
| | | | | | | | | + 8–9 unidentified mar |
| 33 | T | 22 | 7 | 69–89,MAKA | | *1,1,3,4,4, | mar 1 | der(1)?t(1;2)(q25;p25) |
| | | | 8 | 41–47,NCA | | 5,6,6,8,8, | 2 | der(2)?t(1;2)(q25;p25) |
| | | | 18 | 46,XY | | 9,9,10,10, | 3 | del(11)(q23) |
| | | | | | | 13,13,14,14, | | (mar 1–3 two copies per |
| | | | | | | 15,15,16,16, | | cell) |
| | | | | | | 17,17,18 | | + 9–24 unidentified mar |
| 34 | T | 35–38 | 9 | 41–47,NCA | | | | |
| | | | 41 | 46,XY | | | | |
| 35 | T | 9–15 | 20 | (33,35)42–47,MAKA | | | mar 1 | der(2)t(2;?)(p23;?) |
| | | | 6 | (60)89–93,MAKA | | | 2 | del(3)(p?13p?21) |
| | | | 5 | 39–46,NCA | | | 3 | 3p- |
| | | | 7 | 46,XY | | | 4 | der(3)t(3;?)(q?13;?) |
| | | | 2 | 92,XXYY | | | 5 | del(6)(q11) |
| | | | | | | | 6 | t(22;?)(q?13;?) |
| | | | | | | | | + 1–2 unidentified mar |
| 36 | T | 7–20 | 24 | 47–50,XY,+X,+Y,+i(5p),+i(5p)[c] | | | | |
| | | | 8 | 93–100,XXYY,+X,+X,+Y,+Y,+i(5p), +i(5p), +i(5p), +i(5p) | | | | |
| | | | 13 | 46,XY | | | | |
| | PL | 0–17 | | NR | | | | |
| 37 | T | 0–15 | | NR | | | | |
| 38 | T | 0–38 | | NR | | | | |
| | PL | 31–34 | 11 | 41–91,NCA | | | | |
| | | | 17 | 46;XX | | | | |

T, tumour; PL, pleural effusion; MAKA, major karyotype abnormality; clonal abnormalities present in MAKA are listed beside; NCA, non clonal abnormality; NR, no result; *compared with normal tetraploid karyotype.
[a]Figure 1a; [b]Figure 1b; [c]Figure 2.

the stemline. Four patients showed a stemline with a chromosome number in the triploid–tetraploid range. The overall pattern of the cytogenetic findings in the 25 mesotheliomas with clonal abnormalities was complex. Gain and loss of chromosomal material, unbalanced translocations, unidentified markers and clonal evolution were common findings (Figure 1). Many unidentified markers were present in tumour cells from several patients so the interpretation of true chromosomal losses was difficult. Three patients had quite simple karyotypic changes: 48,XX,+5,+7 (patient no. 16), 46,XY,t(9;11)/+21,t(14q;17q)/t(5;13) (three different clones, patient no. 31) and 50,XY,+X,+Y,+i(5p),+i(5p) (stemline of patient no. 36, Figure 2). Clonal numerical chromosome changes including partial or total chromosomal gains and losses are summarised in Figure 3. Distribution of breakpoints of the clonal structural changes on different chromosomes is demonstrated in Figure 4.

**Figure 1** G-banded karyotypes including complex chromosomal changes of tumour cells from patient nos 20 (**a**) and 32 (**b**). A description of the marker chromosomes is given in Table II. The karyotype from patient no. 32 includes, e.g., partial loss of chromosome 1, a breakpoint at 1p13 and loss of chromosome 4. These abnormalities were associated with high concentration of asbestos fibres in lung tissue, which in this patient was 160 million fibres per gram of dried tissue.

### *Consistent chromosomal abnormalities*

No chromosomal abnormality specific to mesothelioma was detected in this study. However, certain chromosomes were preferentially involved in karyotypic changes. The most frequent chromosomal loss, partial or total monosomy 22, was detected in 14 of 25 patients (56%). Material from chromosomes 1 and 3 was missing in 12 patients (48%). In eight patients the short arm of chromosome 1 was lost, and seven of them had an overlapping area in 1p22–1p36.3. Deletions of the short arm of chromosome 3 occurred in seven patients, and all had an overlapping area in 3p13–p21. Partial or total loss of chromosomes 9, 14, 15 and 4 was detected in 11, 11, 10 and 9 patients respectively.

Partial or total polysomy 7 was the most frequent chromosomal gain in the tumour cells of the 25 patients, it was detected in 13 patients (52%). Partial polysomy appeared especially in the form of extra copies of the short arm of chromosome 7 (isochromosomes of 7p were detected in three patients and an additional translocation chromosome involving 7p material in one patient). Gain of chromosome 11 was seen in 11 patients, gain of chromosome 5 in eight patients (two of them having isochromosomes (5p)) and gain of chromosome 12 in seven patients. The involvement of the above abnormalities in the karyotypic changes in the 25 mesotheliomas is presented in Table III.

Most breakpoints occurred in chromosomes 1, 3, 2, 9, 11 and 7, in decreasing order of frequency (Figure 4). Breakage in chromosomal bands 1p11, 1p13, 1p22, 1q21, 3p13, 3p21, 6q12, 7q11.2, 21q22 and 22q11.2 occurred in more than two patients. There was a cluster of breakpoints in regions 1p11–p22 (10 patients, Table III), 1q12–q25 (8 patients), 3p11–21 (6 patients) and 7q11.2 (4 patients).

### *The co-existence of different chromosomal abnormalities*

The correlations between the different chromosomal alterations are demonstrated in Figure 5. Two groups can be separated: a group of chromosomal gains accompanying partial or total polysomy 7, and a group of chromosomal losses accompanying partial or total loss of chromosome 3 or a breakpoint at 1p11–p22. In the first group partial or total polysomy 7 tended to occur together with polysomies 5, 11 and 12 (for each, $P<0.01$). Six of the seven cases that had polysomy 12 had also gained chromosome 11 ($P<0.001$). In the second group, the most statistically significant correlations ($P<0.0001$) were detected between a breakpoint at

**Figure 2** **a**, A G-banded karyotype 50,XY,+X,+Y,+i(5p),+i(5p) from patient no. 36. **b**, A partial R-banded karyotype of B-group chromosomes and isochromosomes (5p).

**Figure 3** Chromosomal gain (left) and loss (right) of the 25 cases with clonal chromosomal abnormalities. Single or combined lines represent one patient. Intact lines are from patients nos 1, 3, 5, 6, 8, 9, 10, 12, 13, 14, 16, 17, 18, 21, 23, 25, 26, 27 and 29 (Tiainen et al., 1988); dotted lines are from patients nos 20, 31, 32, 33, 35 and 36.

1p11–p22 and −14; 1p11–p22 and −15; −1 and −4; −1 and −9; −3 and del(3p); −3 and −9; −3 and −22; and between −4 and −9 (− denoting partial or total loss). No significant associations could be demonstrated between 'chromosome 7 group abnormalities' and 'chromosome 1 and 3 group abnormalities'. On the contrary, there was a significant negative correlation between chromosome 5 and chromosome 3 abnormalities and between chromosome 12 and chromosome 3 abnormalities ($P < 0.01$).

### *Correlations of cytogenetic findings, the content of asbestos fibres in lung tissue and survival*

The following cytogenetic findings were evaluated for correlations with the quantitative data of asbestos fibre content in the corresponding lung tissue and survival: presence of clonal chromosomal abnormalities, non-clonal abnormalities and normal karyotypes (Table I), and the presence of each chromosomal abnormality presented in Table III. As partial poly-

**Figure 4** Distribution of chromosomal breakpoints of cases with clonal abnormalities. Each star and each line (patients nos 1, 3, 5, 6, 8, 9, 10, 12, 13, 14, 16, 17, 18, 21, 23, 25, 26, 27 and 29 (Tiainen *et al.*, 1988)) and each dot and each dotted line (patients nos 20, 31, 32, 33, 35 and 36) represent one breakpoint (lines indicate breakpoints that could not be determined exactly).

**Figure 5** Co-existence of different chromosome abnormalities. −/+, partial or total chromosome loss or gain; del3p, deletion of the p-arm of chromosome 3; bp1p11−p22, breakpoint at 1p11−p22.

somy 7 appeared especially in the form of extra copies of the p-arms, the significance of polysomy 7 was evaluated by the number of additional copies of 7p.

Partial or total losses of chromosomes 1 and 4, and chromosomal rearrangements involving a breakpoint at 1p11−p22 were consistently accompanied by asbestos fibre concentrations greater than 5 million fibres per gram of dried lung tissue ($P = 0.0001$, $P = 0.003$ and $P = 0.009$ respectively, Figure 1b). In addition, the number of copies of chromosome 7 short arms in the tumour cells correlated inversely with survival ($P = 0.02$). Patients with monosomy 7p or with a normal copy number of 7p:s survived longer than those with 1−4 additional 7p:s, and the more copies of chromosome 7 p-arms that were present in the tumour cells the worse was the prognosis (Figure 6).

**Table III** Consistent chromosome changes in 25 mesotheliomas with clonal abnormalities

| | *Chromosome abnormalities* | | | | | | | | | | | | |
|---|---|---|---|---|---|---|---|---|---|---|---|---|---|
| *Patient no.* | $-1^a$ | *Breakpoint 1p11−p22* | $-3^a$ | *del(3p)* | $-4^a$ | $+5^b$ | $+7^b$ | $-9^a$ | $+11^b$ | $+12^b$ | $-14^a$ | $-15^a$ | $-22^a$ |
| 1 | + | + | − | − | + | − | − | + | + | + | + | + | + |
| 3 | + | + | + | + | + | − | − | + | − | − | + | + | + |
| 5 | − | + | − | − | − | − | − | − | − | − | + | + | + |
| 6 | − | + | − | − | − | + | +(1) | − | − | + | − | + | − |
| 8 | − | − | − | − | − | + | +(3) | − | + | + | − | − | − |
| 9 | + | − | − | − | − | − | +(1) | − | + | + | − | − | − |
| 10 | − | + | + | − | − | − | +(3) | − | + | − | + | + | + |
| 12 | + | + | + | − | + | − | − | + | − | − | + | + | + |
| 13 | + | + | + | − | + | − | − | + | − | − | + | − | − |
| 14 | − | − | − | − | − | − | +(1) | − | − | − | − | − | + |
| 16 | − | − | − | − | − | + | +(1) | − | − | − | − | − | − |
| 17 | − | − | + | − | − | − | +(2) | + | + | − | − | − | + |
| 18 | + | − | − | − | − | + | +(1) | − | − | − | − | − | + |
| 20 | + | + | + | + | + | − | − | + | + | − | + | + | + |
| 21 | + | − | + | + | − | − | +(2) | + | − | − | − | − | + |
| 23 | + | − | − | − | + | + | +(2) | + | + | + | − | + | − |
| 25 | + | + | + | + | + | − | − | + | − | − | + | − | + |
| 26 | − | − | + | + | − | − | +(1) | + | + | − | + | − | + |
| 27 | + | − | + | − | + | − | − | − | − | − | − | − | + |
| 29 | − | − | − | − | − | + | +(4) | − | + | + | − | + | − |
| 31 | − | − | − | − | − | − | − | − | − | − | + | − | − |
| 32 | + | + | + | + | + | − | − | + | + | − | + | + | − |
| 33 | − | − | − | − | − | + | +(1) | − | + | + | − | − | − |
| 35 | − | − | + | + | − | − | − | − | − | − | − | − | + |
| 36 | − | − | − | − | − | + | − | − | − | − | − | − | − |
| Total+ | 12 | 10 | 12 | 7 | 9 | 8 | 13 | 11 | 11 | 7 | 11 | 10 | 14 |

[a]Partial or total monosomy; [b]partial or total polysomy; number of extra copies of 7p in parentheses.

**Figure 6** Correlation between the number of copies of chromosome 7 p-arms and survival ($P = 0.02$). **a**, Four extra 7p:s (patient no. 29); **b**, three extra 7p:s (patients nos 8 and 10); **c**, two extra 7p:s (patients nos 17, 21 and 23); **d**, one extra 7p (patients nos 6, 9, 14, 16, 18, 26 and 33); **e**, normal number of 7p:s (patients nos 1, 2, 4, 5, 11, 12, 15, 19, 20, 22, 24, 25, 27, 31, 34, 35, 36 and 38); and **f**, loss of a copy of 7p (patients nos 3, 13 and 32).

### Discussion

In the present study the cytogenetic data were obtained mainly from specimens from the primary tumours received before treatment of 34 patients with malignant pleural mesothelioma. The histological diagnosis and subtyping were based on the same specimens. Karyotypic analyses of the 25 mesotheliomas with clonal chromosome abnormalities revealed numerous and often complex chromosomal changes, and heterogeneity between different cases and within a single case. The following partial or total chromosomal gains (+) and losses (−) were found most frequently: −22; +7, −1, −3, −9, +11, −14, −15, −4, +5 and +12 (in decreasing order of frequency). Clustering of breakpoints was detected in regions 1p11−22, 1q12−q25, 3p11−21 and 7q11.2, of which 1p11−p22 was the most common. These findings are partly confirmed by other studies. Deletions of the short arm of chromosome 3 have been the most frequent abnormalities in the 25 cases reported by Ayraud (1975), Mark (1978), Wake *et al.* (1981), Gibas *et al.* (1986), Stenman *et al.* (1986), Bello *et al.* (1987) and Popescu *et al.* (1988). Structural abnormalities involving a breakpoint at 1p11−p22, and both structural and numerical changes involving chromosome 7 have also been detected commonly in those studies. In a recent study of chromosomal changes in 38 mesotheliomas (Hagemeijer *et al.*, 1988) (abnormal clones in 26 cases) consistent involvement of chromosomes 1, 3, 6, 9 and 13 in structural changes and chromosomes 4 and 22 in losses and chromosomes 5, 7 and 20 in gains was found.

Our analysis of the co-existence of different chromosome abnormalities showed that two groups could be separated: chromosome 5, 7, 11 and 12 gains in the first group, and chromosome 1, 3, 4, 9, 14, 15 and 22 losses and a breakpoint at 1p11−p22 in the second. Such correlations at the chromosomal level may reflect correlations at the molecular level: co-operation of different oncogenes or growth factor genes, loss of suppressor genes, gene dosage effect or gene position effect. It is interesting that NRAS and two NRAS-like oncogenes are located in chromosomal regions frequently involved in chromosomal changes in mesotheliomas: 1p13 and/or 1p22, 9p and 22 (Human Gene Mapping 9.5, 1988). The possible role of NRAS in mesothelioma is not yet known. Several oncogenes, like ERBB (EGFR), which has been shown to be amplified in some tumours (Hollstein *et al.*, 1988), and the multiple drug resistance gene (P-glycoprotein gene), the overexpression of which has been detected in drug resistant cell lines (Lemontt *et al.*, 1988), are located on chromosome 7 (Human Gene Mapping 9.5, 1988). Elevation of platelet-derived growth factor (PDGF) A- and B-chain expression has been detected in human mesothelioma cell lines (Gerwin *et al.*, 1987; Versnel *et al.*, 1988b). The PDGF A-chain gene is located on chromosome 7 (Bonthron *et al.*, 1988) and the PDGF B-chain gene is on chromosome 22 (Human Gene Mapping 9.5, 1988). The PDGF B-chain receptor has also been shown to be overexpressed in mesothelioma cell lines suggesting possible autocrine growth of mesothelioma cells by this growth factor (Versnel *et al.*, 1988*a*). The PDGF receptor has been mapped to chromosome 5 (Human Gene Mapping 9.5, 1988).

The various chromosomal abnormalities in our study and their complex relations may reflect the fact that the tumours were diagnosed and studied at an advanced stage of their evolution. The primary chromosomal abnormalities could have become masked by several secondary changes related to tumour progression or ageing. However, three patients in this study had quite simple karyotypic changes: patient no. 16: 48,XX,+5,+7; patient no. 31: 46,XY,t(9;11)/+21,t(14 g;17 g)/t(5;13) (three different clones); and patient no. 36: 50,XY,+X,+Y,+i(5p),+i(5p). Patients nos 6 and 8 also had simple clones (45,XY,−Y and 47,XY,+Y respectively) in specimens obtained before treatment from their tumours, but quite different clones involving complex chromosomal abnormalities in other specimens obtained also before treatment from the same tumours. It may be possible that in patients nos 16, 31 and 36, as well as in the patients with normal karyotypes and/or nonclonal abnormalities, complicated clones were also present but not detected by our current cytogenetic analysis.

Distinguishing mesothelioma from carcinoma metastatic to the pleura remains a major problem since there is no specific stain or test to identify mesothelioma cells and only few carcinomas have intracellular mucin that is diagnostic to adenocarcinoma. Several recently described monoclonal antibodies may facilitate accurate diagnosis (Lee *et al.*, 1986; Anderson *et al.*, 1987).

The consistent chromosomal abnormalities in mesothelioma shown in this and in other studies do not, at present, have diagnositic value. Several other solid tumours share similar patterns of chromosomal alterations. In a study of 48 different specimens from solid tumours of 10 different tissues, especially duplication of chromosome 7 and structural abnormalities involving chromosomes 1, 3, 7 and 11 were preferentially present in the karyotypic changes (Teyssier, 1987). The same abnormalities are also described in adenocarcinoma of the lung (Rey *et al.*, 1987; Fan & Li, 1987) the pleural metastases of which may be difficult to distinguish histologically and cytologically from mesothelioma. Breakpoints at 1p12−p22 are frequently seen in malignant melanoma (Balaban *et al.*, 1986) and structural changes of chromosome 1 are common in several tumours (Brito-Babapulle & Atkin, 1981). Deletions of 3p are specific abnormalities in small cell carcinomas of the lung (Whang-Peng *et al.*, 1982) and renal cell carcinomas (Kovacs *et al.*, 1987). Extra copies of chromosome 7 are frequent in malignant melanoma (Balaban *et al.*, 1986), bladder cancer (Sandberg, 1986) and malignant glioma (Bigner *et al.*, 1988). Monosomy 22 is characteristic to meningioma (Zang, 1982).

Some of the consistent chromosomal abnormalities in mesothelioma in our study showed a correlation with the asbestos fibre burden of the corresponding lung. Partial or total losses of chromosomes 1 and 4, and translocations and deletions involving a breakpoint at 1p11−p22 correlated with high content of asbestos fibres in lung tissue. In asbestos-induced rat mesotheliomas (Libbus & Craighead, 1988) and in Syrian hamster cell lines transformed by asbestos (Oshimura *et al.*, 1986) non-random karyotypic changes have been described. It is not yet demonstrated that asbestos induces specific chromosome changes in human mesothelial cells. So far we are aware of only one report of chromosomal defects in human mesothelial cells exposed to asbestos (Lechner *et al.*, 1985). Losses of chromosomes 11 and 21, dicentric chromosomes, double minute chromosomes and other markers were described. It is possible that asbestos fibres cause chromosomal abnormalities at random sites but that cells with chromosomal rearrangements in the region 1p11–p22 or losses of chromosomes 1 or 4 have a selective advantage in tumour progression. The possible relation between the NRAS oncogene located in 1p13 and/or 1p22 and exposure to asbestos remains unknown. It is also noteworthy that the 1p11–p22 region contains several fragile sites (Human Gene Mapping 9.5, 1988) where breakage could be induced more easily.

There was a statistically significant correlation between the number of copies of chromosome 7 p-arms and the prognosis. The more copies of 7p:s that were present in the tumour cells the shorter was survival. Results of the different multimodality treatment programs used in our patient series have been reported elsewhere (Holsti & Mattson, 1988). None of the therapies was efficient, and there was no significant difference between the groups. Trisomy 7 (or i(5p)) has been suggested to be associated with increased aggressiveness of a tumour in bladder cancer (Sandberg, 1986). Additionally, extra copies of chromosome 7 p-arms have been described in advanced malignant melanomas (Balaban *et al.*, 1986). Chromosome 7 includes several protooncogenes and growth factor genes the amplification of which could affect the tumour's behaviour.

In this study no diagnostic marker of mesothelioma was identified by our cytogenetic analysis. Instead, correlations between chromosome abnormalities, asbestos exposure and survival were demonstrated. Further studies are, however, needed to confirm whether the copy number of chromosome 7 short arms can be considered a prognostic factor in mesothelioma. When more cytogenetic and molecular genetic data about mesothelioma accumulate mesothelioma-specific genetic alterations may be detected and new clinical correlations will be identified.

This work was supported partly by the Finnish Cancer Society and the Sigfrid Jusélius Foundation. We thank Miss Paula Pasuri for technical assistance and Ragnar Wessman MSc for preparing the Figure 5.

### References

ANDERSON, T.M., HOLMES, E.C., KOSAKA, C.J., CHENG, L. & SAXTON, R. (1987). Monoclonal antibodies to human malignant mesothelioma. *J. Clin. Immunol.*, **7**, 254.

ANTMAN, K.H. (1980). Current concepts. Malignant mesothelioma. *N. Engl. J. Med.*, **303**, 200.

AYRAUD, N. (1975). Identification par denaturation thermique menagée des anomalies chromosomiques observées dans six tumeurs metastatiques humaines. *Biomedicine*, **23**, 423.

BALABAN, G.B., HERLYN, M., CLARC JR., W.H. & NOWELL, P.C. (1986). Karyotypic evolution in human malignant melanoma. *Cancer Genet. Cytogenet.*, **19**, 113.

BATTIFORA, H. & KOPINSKI, M.I. (1985). Distinction of mesothelioma from adenocarcinoma, an immunohistochemical approach. *Cancer*, **55**, 1679.

BELLO, M.J., REY, J.A., AVILES, M.J., AREVALO, M. & BENITEZ, J. (1987). Cytogenetic findings in an effusion secondary from pleural mesothelioma. *Cancer Genet. Cytogenet.*, **29**, 75.

BIGNER, S.H., MARK, J., BURGER, P.C. & 4 others (1988). Specific chromosomal abnormalities in malignant human gliomas. *Cancer Res.*, **88**, 405.

BONTHRON, D.T., MORTON, C.C., ORKIN, S.H. & COLLINS, T. (1988). Platelet-derived growth factor A chain: gene structure, chromosomal location, and basis for alternative mRNA splicing. *Proc. Natl Acad. Sci. USA.*, **85**, 1492.

BRITO-BABAPULLE, V. & ATKIN, N.B. (1981). Break points in chromosome 1 abnormalities of 218 human neoplasms. *Cancer Genet. Cytogenet.*, **4**, 215.

BROWNE, K. (1986). Mesothelioma registry data. *Lancet*, **i**, 167.

BURNS, T.R., GREENBERG, S.D., MACE, M.L. & JOHNSON, E.H. (1985). Ultrastructural diagnosis of epithelial malignant mesothelioma. *Cancer*, **56**, 2036.

CORSON, J.M. (1987). Pathology of malignant mesothelioma. In *Asbestos-related Malignancy*, Antman, K. & Aisner, J. (eds) p. 179. Grune & Stratton: Orlando, Florida.

DIXON, W.J. (ed.) (1985). *BMDP Statistical Software*. University of California Press: Los Angeles.

FAN, Y.S. & LI, P. (1987). Cytogenetic studies of four human lung adenocarcinoma cell lines. *Cancer Genet. Cytogenet.*, **26**, 317.

GERWIN, B.I., LECHNER, J.F., REDDEL, R.R. & 4 others (1987). Comparison of production of transforming growth factor-$\beta$ and platelet-derived growth factor by normal human mesothelial cells and mesothelioma cell lines. *Cancer Res.*, **47**, 6180.

GIBAS, Z., LI, F.P., ANTMAN, K.H., BERNAL, S., STAHEL, R. & SANDBERG, A.A. (1986). Chromosome changes in malignant mesothelioma. *Cancer Genet. Cytogenet.*, **20**, 191.

HAGEMEIJER, A., VERSNEL, M.A., SCHOENMAKER, E., MORET, M. & BOUTS, M.J. (1988). Cytogenetic analysis of malignant mesotheliomas. Meeting abstract no. 124. *1st European Workshop on Cytogenetics and Molecular Genetics of Human Solid Tumours.* 13–15 October 1988, Dijon, France.

HEIM, S. & MITELMAN, F. (1987). *Cancer Cytogenetics*. A.R. Liss: New York.

HOLLSTEIN, M.C., SMITS, A.M., GALIANA, C. & 5 others (1988). Amplification of epidermal growth factor receptor gene but no evidence of *ras* mutations in primary human esophageal cancers. *Cancer Res.*, **48**, 5119.

HOLSTI, L. & MATTSON, K. (1988). Hemithorax irradiation in the treatment of pleural mesothelioma. *Proceedings of the Fifth Varian European Clinac Users Meeting*, 12–14 February 1987, Flims, Switzerland, p. 166.

HUMAN GENE MAPPING 9.5 (1988): Update to the Ninth International Workshop on Human Gene Mapping. *Cytogenet. Cell Genet.*, **49**, 1.

KOVACS, G., SZUCS, S., DE RIESE, W. & BAUMGÄRTEL, H. (1987). Specific chromosome aberration in human renal cell carcinoma. *Int. J. Cancer*, **40**, 171.

LECHNER, J.F., TOKIWA, T., LA VECK, M. & 5 others (1985). Asbestos-associated chromosomal changes in human mesothelial cells. *Proc. Natl Acad. Sci. USA.*, **82**, 3884.

LEE, I., RADOSEVICH, J.A., CHEJFEC, G. & 4 others (1986). Malignant mesotheliomas. Improved differential diagnosis from lung adenocarcinomas using monoclonal antibodies 44–3A6 and 624A12. *Am. J. Pathol.*, **123**, 497.

LEMONTT, J.F., AZZARIA, M. & GROS, P. (1988). Increased *mdr* gene expression and decreased drug accumulation in multidrug-resistant human melanoma cells. *Cancer Res.*, **48**, 6348.

LIBBUS, B.L. & CRAIGHEAD, J.E. (1988). Chromosomal translocations with specific breakpoints in asbestos-induced rat mesotheliomas. *Cancer Res.*, **48**, 6455.

MARK, J. (1978). Monosomy 14, monosomy 22 and 13q-. Three chromosomal abnormalities observed in cells of two malignant mesotheliomas studied by banding techniques. *Acta Cytol.*, **22**, 398.

MATTSON, K., HOLSTI, L.R., TAMMILEHTO, L. & 5 others (1989). Multimodality treatment programs using high-dose hemithorax irradiation for malignant pleural mesothelioma. *Int. J. Radiat. Oncol. Biol. Phys.* (in the press).

OSHIMURA, M., HESTERBERG, T.W. & BARRET, J.C. (1986). An early, nonrandom karyotypic change in immortal Syrian hamster cell lines transformed by asbestos: trisomy of chromosome 11. *Cancer Genet. Cytogenet.*, **22**, 225.

POPESCU, N.C., CHAHINIAN, A.P. & DIPAOLO, J.A. (1988). Nonrandom chromosome alterations in human malignant mesothelioma. *Cancer Res.*, **48**, 142.

REY, J.A., BELLO, M.J., DE CAMPOS, J.M., KUSAK, M.E., MORENO, S. & BENITEZ, J. (1987). Deletion 3p in two lung adenocarcinomas metastatic to the brain. *Cancer Genet. Cytogenet.*, **25**, 355.

SANDBERG, A.A. (1986). Chromosome changes in bladder cancer: clinical and other correlations. *Cancer Genet. Cytogenet.*, **19**, 163.

STENMAN, G., OLOFSSON, K., MÅNSSON, T., HAGMAR, B. & MARK, J. (1986). Chromosomes and chromosomal evolution in human mesotheliomas as reflected in sequential analyses of two cases. *Hereditas*, **105**, 233.

TEYSSIER, J.R. (1987). Nonrandom chromosomal changes in human solid tumors: application of an improved culture method. *J. Natl Cancer Inst.*, **79**, 1189.

TIAINEN, M., TAMMILEHTO, L., MATTSON, K. & KNUUTILA, S. (1988). Nonrandom chromosomal abnormalities in malignant pleural mesothelioma. *Cancer Genet. Cytogenet.*, **33**, 251.

TUOMI, T., SEGERBERG-KONTTINEN, M., TAMMILEHTO, L., TOSSAVAINEN, A. & VANHALA, E. (1989). Mineral fiber concentration in lung tissue of mesothelioma patients in Finland. *Am. J. Indust. Med.* (in the press).

VERSNEL, M.A., BOUTS, M.J., HOOGSTEDEN, H.C., VAN DER KWAST, T.H. & HAGEMEIJER, A. (1988*a*). Expression of the PDGF B- (c-*sis*) and PDGF A-chain genes and the PDGF B-type receptor in human malignant mesothelioma cell lines. Meeting abstract no. 133. *1st European Workshop on Cytogenetics and Molecular Genetics of Human Solid Tumors*. 13–15 October 1988, Dijon, France.

VERSNEL, M.A., HAGEMEIJER, A., BOUTS, M.J., VAN DER KWAST, T.H. & HOOGSTEDEN, H.C. (1988*b*). Expression of c-sis (PDGF B-chain) and PDGF A-chain genes in ten human malignant mesothelioma cell lines derived from primary and metastatic tumors. *Oncogene*, **2**, 601.

WAKE, N., SLOCUM, H.K., RUSTUM, Y.M., MATSUI, S. & SANDBERG, A.A. (1981). Chromosomes and causation of human cancer and leukemia. XLIV. A method for chromosome analysis of solid tumours. *Cancer Genet. Cytogenet.*, **3**, 1.

WHANG-PENG, J., BUNN JR., P.A., KAO-SHAN, C.S. & 4 others (1982). A nonrandom chromosomal abnormality, del3p(14–23), in human small cell lung cancer (SCLC). *Cancer Genet. Cytogenet.*, **6**, 119.

ZANG, K.D. (1982). Cytological and cytogenetical studies on human meningioma. *Cancer Genet. Cytogenet.*, **6**, 249.